# Effects of estradiol and FSH on maturation of the testis in the hypogonadal (hpg) mouse

**DOI:** 10.1186/1477-7827-6-4

**Published:** 2008-01-29

**Authors:** Helen Baines, Margaret O Nwagwu, Graham R Hastie, Roman A Wiles, Terry M Mayhew, Francis JP Ebling

**Affiliations:** 1 School of Biomedical Sciences, University of Nottingham Medical School, Queen's Medical Centre, Nottingham NG7 2UH, UK

## Abstract

**Background:**

The hypogonadal (hpg) mouse is widely used as an animal model with which to investigate the endocrine regulation of spermatogenesis. Chronic treatment of these GnRH-deficient mice with estradiol is known to induce testicular maturation and restore qualitatively normal spermatogenesis. The aim of the current studies was to investigate whether these effects of estradiol are direct effects in the testis, or indirect actions via paradoxical stimulation of FSH secretion from the pituitary gland.

**Methods:**

Initially, Western blot and immunohistochemistry were used to analyse tissues from hpg mice to identify potential sites of action of estradiol. In the main study, hpg mice were treated for 50 days with either an estradiol implant or daily injections of recombinant human FSH, or a combination of both, to determine whether estradiol would have an additive or synergistic effect with FSH on testis development, as assessed by histological analysis and stereological quantification of Leydig, Sertoli and germ cell proliferation.

**Results:**

Western blot analysis revealed ERα immunoreactive bands of appropriate molecular weight in extracts of testis and pituitary glands from hpg mice, and immunohistochemical studies confirmed ERα in nuclei of anterior pituitary cells and Leydig and peritubular cells in hpg mice. Histological and morphometric analyses revealed that estradiol treatment alone was as effective as FSH in promoting Sertoli cell production and proliferation of the seminiferous epithelium, resulting in the production of elongating spermatids. Combined estradiol and FSH treatment did not produce a greater effect than either treatment alone, though an increased dose of FSH significantly increased seminiferous tubule volume and testis weight and increase Sertoli cell numbers further within the same time frame. In contrast, estradiol caused substantial increases in the wet weight of the seminal vesicles, whereas FSH was without effect on this tissue, and did not augment the actions of estradiol.

**Conclusion:**

As ERalpha receptor is abundantly expressed in the pituitary gland of hpg mice, and estradiol did not exert effects on testis development over and above those of FSH, we conclude that the action of estradiol on testis development in *hpg *mice is predominantly via the stimulation of pituitary FSH release.

## Background

Traditionally, the regulation of mammalian spermatogenesis has been viewed as being dependent upon the interactions of gonadotrophins and androgens, however, recent observations in experimental rodents and man have indicated additional roles for estrogen in this process [[1,2] for reviews]. Human studies have demonstrated the presence of aromatase in the testis together with the expression of two splice variants of ERβ in somatic and germ cell compartments, though expression of ERα appears to be restricted to the efferent ductules [3]. The importance of estrogen in male reproduction is illustrated by the infertility displayed by men with inactivating mutations in the gene encoding aromatase or ERα [4]. Mice have been widely used to investigate the function of estrogens in male reproduction, indeed the targeted deletion of estrogen receptors [5] and aromatase [6] provided the first evidence for a role for estrogen in sperm production. In contrast to primates, ERα is expressed abundantly in the Leydig cells of mice, though it seems likely that the infertility in male ERα knockout mice results from inadequate fluid resorption in the efferent ductules rather than a primary deficit in spematogenesis [7]. The function of ERα in the interstitial cells of the rodent testis is not understood.

We and other research groups have exploited the hypogonadal (*hpg*) mouse [8], which cannot produce mature GnRH decapeptide due to a truncation in the GnRH gene, as a model to study the role of estrogen in male reproductive function [9,10]. *Hpg *mice are infertile because they do not produce gonadotropin and, in consequence, testis development does not proceed beyond the neonatal stage [11]. It is well established that treatment of *hpg *mice with LH can stimulate steroidogenesis [12] and treatment with FSH and androgens can induce qualitatively normal spermatogenesis [13,14]. However, we have also observed that chronic estradiol treatment alone can induce spermatogenesis in *hpg *mice [9,15,16], though this is associated with paradoxical increases in pituitary and serum FSH concentrations. The importance of this estrogen-induced increase in FSH secretion is not known: it has not been established whether estradiol acting directly at the level of the testis is sufficient to produce qualitative spermatogenesis in *hpg *mice or whether the effects of estradiol in inducing spermatogenesis are more indirect, occurring via the rise in FSH production.

This aim of the study was to investigate the relative contribution of direct (testis) and indirect (pituitary) actions of estradiol in stimulating spermatogenesis. The initial objective of the study was to use immunohistochemistry and Western blotting to localise ERα in the pituitary gland and testis of *hpg *mice, in order to identify putative sites of estrogenic action in promoting spermatogenesis in this mutant strain. The main objective was to measure the qualitative and quantitative effects of estradiol on testis development and spermatogenesis in *hpg *mice in the presence and absence of rhFSH. If combined estradiol and rhFSH treatment proved to be more effective than either of these treatments alone (i.e. there was a synergistic effect), we would conclude that these hormones exert separate actions. If, however, combined estradiol and rhFSH treatment were to be only as efficacious as would be expected from a higher rhFSH dose (i.e. an additive effect) then we would conclude that estradiol most likely acts via stimulation of FSH secretion.

## Methods

### General

All animal procedures were approved by the University of Nottingham Local Ethical Review Committee and carried out in accordance with the Animals Scientific Procedures Act (UK) 1986 (project licence PPL 40/2372). Laboratory Animal Science Association (LASA) guidelines were followed for administration of substances [17]. Adult *hpg *male mice were identified by their micropenis, reduced anogenital distance and small scrotal sac.

### Western blot procedure

Pituitary glands and testes from *hpg *and age-matched littermates mice (approximately 135 days old) were placed in phosphate-buffered saline at pH7.4 (PBS) containing a protease inhibitor. Tissues were sonicated on ice for 3 × 10 sec, centrifuged at 1300 RPM for 5 min and the supernatant extracted and stored at -20°C until used. Testis and pituitary protein were incubated at 100°C for 5 mins in the presence of 5% mercaptoethanol. Samples were then immediately cooled on ice and loaded onto the gel. Protein samples were separated on a 10% SDS gel and transferred to a nitrocellulose membrane (Amersham). Membranes were blocked with 5% milk (Marvel) in PBS at 25°C for 1 hour before incubation overnight at 4°C with a polyclonal antisera raised in rabbit recognising ERα (1:50 dilution of MC-20, Santa Cruz, USA). After overnight incubation membranes were washed in PBS, then PBS/Tween 20, and subsequently incubated with 1:2000 anti-rabbit IgG peroxidase conjugate (Sigma, Poole, UK). Visualization of the protein was achieved using the supersignal system (Pierce, USA). The molecular weight of the protein was estimated by comparing its mobility with standard marker proteins of known molecular weights. The relative amounts of the protein indicated by the intensity of the bands was evaluated by analysing scans of the film and performing densitometry using the NIH Image computer package.

### Immunohistochemistry

Testis and pituitary tissue was fixed by immersion in 4% paraformaldehyde, then embedded in paraffin wax so that 5 um sections could be cut on a microtome. Mounted sections were then placed in xylene to remove the paraffin wax, and placed in decreasing concentrations of ethanol and then tap water to rehydrate the tissue. The pituitary and testis sections were subject to antigen retrieval in 0.01 M citrate buffer pH6 by microwaving at full power (650 W) for 2 × 5 mins. For the studies on estrogen receptor distribution, sections were washed in phosphate buffered saline (PBS) buffer and blocked for endogenous peroxidase with 3% hydrogen peroxide. Following rinsing in PBS, samples were blocked with normal goat serum (Vector Labs, Peterborough, UK) then incubated overnight at 4°C with the previously validated rabbit polyclonal ERα antiserum (MC-20, Santa Cruz) at a dilution of 1:50. Sections were washed in PBS and incubated with a biotinylated anti-rabbit IgG at 25°C for 1 hour followed by an avidin biotin horseradish peroxidase complex (Elite ABC: Vector Labs, Peterborough, UK). Immunoreactivity was revealed by incubation with diaminobenzidine (DAB) and the reaction stopped by immersing sections in tap water. Sections were counterstained with eosin. After dehydration through increasing concentration of ethanol, sections were mounted using DePeX mounting medium (Sigma, Poole, UK).

For the studies on where the expression of the steroidogenic enzymes 3β hydroxysteroid dehydrogenase (3βHSD) was used to identify Leydig cells, a similar protocol was followed but omitting the pre-treatment with 3% hydrogen peroxide. Sections were incubated overnight in a polyclonal rabbit anti-3βHSD antiserum (dilution of 1:1000) donated by Prof Ian Mason, University of Edinburgh, and previously validated for use in rodents [18,19]. Sections were washed in PBS and incubated with a biotinylated anti-rabbit IgG at 25°C for 1 hour followed by a fluorescein-avidin complex (Vector Labs). After washes in PBS then distilled water, sections were coverslipped using Vectashield (Vector Labs), sealed with nail polish and viewed under a Leica DMRB Fluorecence microscope.

### Experimental protocol: effects of estradiol and rhFSH

Under general anesthesia, male *hpg *mice ranging in age from 3–7 months were implanted with either a subcutaneous capsule containing 2% estradiol (n = 11) or cholesterol (n = 15) as previously described [9]. Subgroups of *hpg *mice bearing estradiol implants were then treated daily with 1 IU rhFSH (n = 6) or vehicle (n = 5) for 50 days. Correspondingly, subgroups of *hpg *mice bearing cholesterol implants were treated daily with 1 IU rhFSH (n = 5) or 5 IU rhFSH (n = 6) or vehicle (n = 5) for 50 days. The doses of rhFSH were based on those used in a previous dose-response study in *hpg *mice and were designed to produce a submaximal (1 IU/day) and a maximal (5 IU/day) effect [14]. On experimental day 50, blood samples were collected by cardiac puncture under terminal anesthesia (sodium pentobarbitone, i.p.) 2 h after the last FSH treatment. Blood was allowed to clot at room temperature before being spun at 13000 rpm and then serum samples were stored at -20°C until assayed. After blood collection, the pituitary, testes, epididymides and seminal vesicles were excised, trimmed of fat and connective tissue and weighed. One testis was placed in Bouin's fixative, and subsequently embedded in paraffin wax so that 5 um sections could be cut on a microtome and stained with haematoxylin-eosin for light microscope analysis.

### Morphometric (stereological) analyses

The primary morphometric aims were to estimate volumes of seminiferous tubules and seminiferous epithelium within testes. To this end, stereological estimates [20,21] were made on tissue images randomised for location and orientation by a multistage systematic uniform random sampling design. Approximately four sections per testis, and six microscopical fields per section, were randomly chosen for analysis. Fields were sampled as images captured on a Leica DM4000B brightfield microscope via OpenLab software (Improvision, Coventry, UK) and printed to a final magnification of ×1000 using a stage micrometer scale as an external calibration standard. Volume densities of testicular ingredients were determined by randomly superimposing a transparent grid comprising 35 test points arranged in a quadratic array. Test points falling on a given testis and its ingredients were summed over all fields from all sections. The total number of points landing on a given ingredient (tubule, lumen, epithelium), divided by the total number of points landing on the testis sections, provided an unbiased estimate of its volume density. The absolute volume of each ingredient per testis was estimated subsequently from the product of the volume density and the processed testicular volume. In order to assess whether changes in tubule volume were attributable to changes in calibre or total length, mean diameters of seminiferous tubules were measured after selecting tubules using an unbiased counting frame [20]. In the case of elliptical profiles, the short axis was measured. To give an indication of the degree of germ cell development, each of the 24 fields per testis collected for stereological analysis was also scored by an observer blind to the experimental treatment to indicate the most mature germ cell type present.

Quantitative assessment of Leydig and Sertoli cell numbers was made in order to obtain relative measures of net cell proliferation. Estimates were obtained by randomly superimposing a forbidden line unbiased counting frame [20] on enlarged photomicrographs of tissues immunostained for 3β-HSD (Leydig cell marker) or on tissue sections stained with haematoxylin and eosin where the nuclei of Sertoli cells could be unequivocally identified by their characteristic location and appearance (eg tripartite nucleolus, [11]). Using this counting frame, the numbers of nuclear profiles per unit area of testis were used to obtain model-based estimates of the numbers of nuclei per unit volume of tissue (N_v_). To this end, we obtained estimates of section thickness (T, always 5 μm) and the mean diameters (D, in μm) of nuclei for each cell type. To estimate the mean nuclear diameter, the diameters of 10 randomly selected nuclei were measured. On the explicit assumption that each cell contains a single nucleus, the total number of specific cell types per testis (N) was then calculated from

N = N_v _× V_testis_

where V_testis _represents testis volume (cm^3^) was calculated by dividing testis mass (g) by tissue density. The latter was taken to be 1.05 g/cm^3 ^[22].

### Hormone assays

Serum FSH concentrations were measured using both a commercially available human FSH ELISA kit (IDS Ltd UK) to determine the levels of recombinant hFSH circulating, and a rat FSH immunoradiometric kit (IDS Ltd UK) in order to estimate the total circulating FSH concentrations (i.e. endogenous and exogenous). FSH concentrations in extracts of mouse pituitary gland, or in untreated wild-type mouse serum, did not dilute in parallel with the rat standards, and there was substantial cross reactivity of rhFSH in the rat IRMA (1 mIU rhFSH reading as approximately 1.5 ng/ml of the rat FSH standard) so all experimental samples were compared at the same dilution in a single assay and must be considered as relative concentrations rather than absolute values. The minimum detection limit in the rat FSH IRMA was 0.2 ng/ml and the intra-assay CV was 2.0%. The minimum detection limit in the human FSH ELISA was 1 mIU/ml and the intra-assay CV was 7.5%.

### Statistical analysis

Data for organ weights were analysed using one-factor ANOVA followed by Tukey's multiple comparison test (Prism v4.0, GraphPad Software, San Diego, CA). FSH values were analysed using Kruskal-Wallis non-parametric tests (also Prism) because when values were below the limit of detection of the assay they were assigned that value for the purposes of statistical analysis.

## Results

### Estrogen receptor immunoreactivity in the pituitary gland and testis

**Figure 1:**
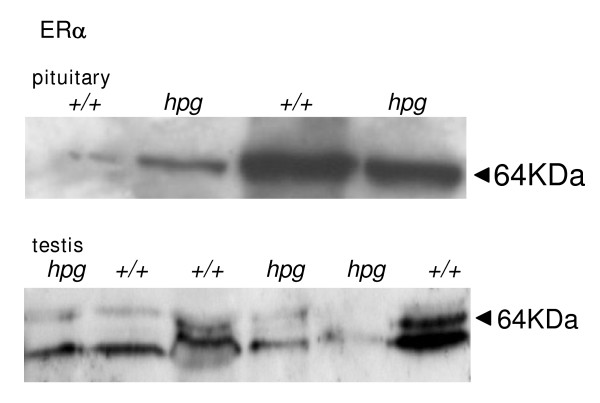
**Western blot analysis of ERα immunoreactivity**. Examples of a Western blot analysis of extracts of pituitary gland (top) and testis (bottom) from hypogonadal (*hpg*) and age-matched wild-type (+/+) mice. Membranes were stained with MC-20 ERα antiserum (Santa Cruz).

**Figure 2:**
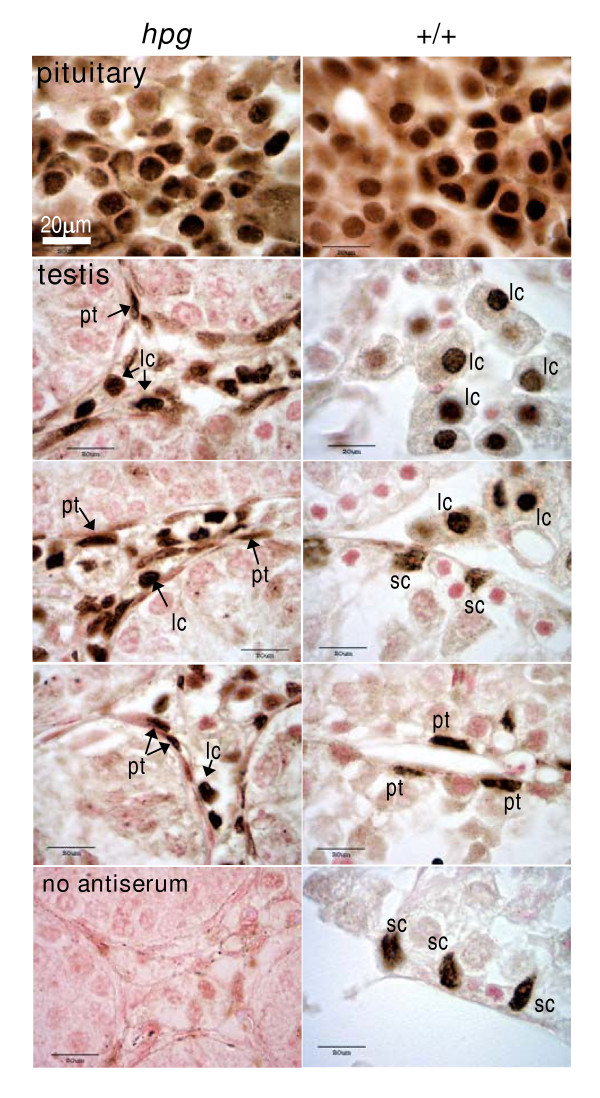
**Immunohistochemical localization of ERα in *hpg *mice**. Examples of immunohistochemical localization of ERα in the pituitary gland (upper panels) or testis of hypogonadal mice (*hpg*, left panels) or age-matched wild-type mice (+/+, right panels). Note that many anterior pituitary cells express nuclear ERα immunoreactivity in both *hpg *and +/+ mice. ERα-ir is abundant in the Leydig cells (lc) and peritubular myoid cells (pt) in the testis of *hpg *mice, and is also present in some Sertoli cells (sc) in wild-type mice. Bottom left panel shows a control study in which the primary antiserum was omitted, the section is from a *hpg *testis. All photomicrographs were taken at the same magnification so the 20 μm scale bar in top left panel applies to all panels

Western blots revealed a clear band of ERα-immunoreactivity in extracts of pituitary tissue from adult *hpg *mice at a molecular weight of approximately 64 KDa (Fig. 1, top). There was no significant difference in abundance comparing samples from *hpg *(n = 4) and wild type (n = 4) mice. ERα-immunoreactive bands were also observed at approximately 64 KDa in extracts of testis from both *hpg *and wild-type mice, plus a slightly lower molecular weight band which is likely to be a degradation artefact (Fig. 1, bottom). No statistical difference in abundance of the two bands between the genotypes was observed (Fig. 1). Immunohistochemistry revealed a clear nuclear localization of ERα in anterior pituitary cells in both *hpg *mice and wild-type litter mates (Fig. 2). ERα-immunoreactivity was prominent in the nuclei of both Leydig cells and peritubular myoid cells in testes from *hpg *mice (Fig. 2, left). Leydif cells in the age-matched wild-type testis also expressed ERα-immunoreactivity (Fig. 2, right), and some peritubular cells also expressed ERα (Fig. 2, right). In addition, in wild-type mice there ws some positive immunostaining of cells adjacent to the basal membrane with an irregular shaped nucleus which are almost certainly Sertoli cells (Fig. 2, right). The immunoreactive signal was absent when the primary antiserum was replaced with rabbit IgG (Fig. 2, bottom left).

### Effects of estradiol and rhFSH on reproductive organ weight

**Figure 3:**
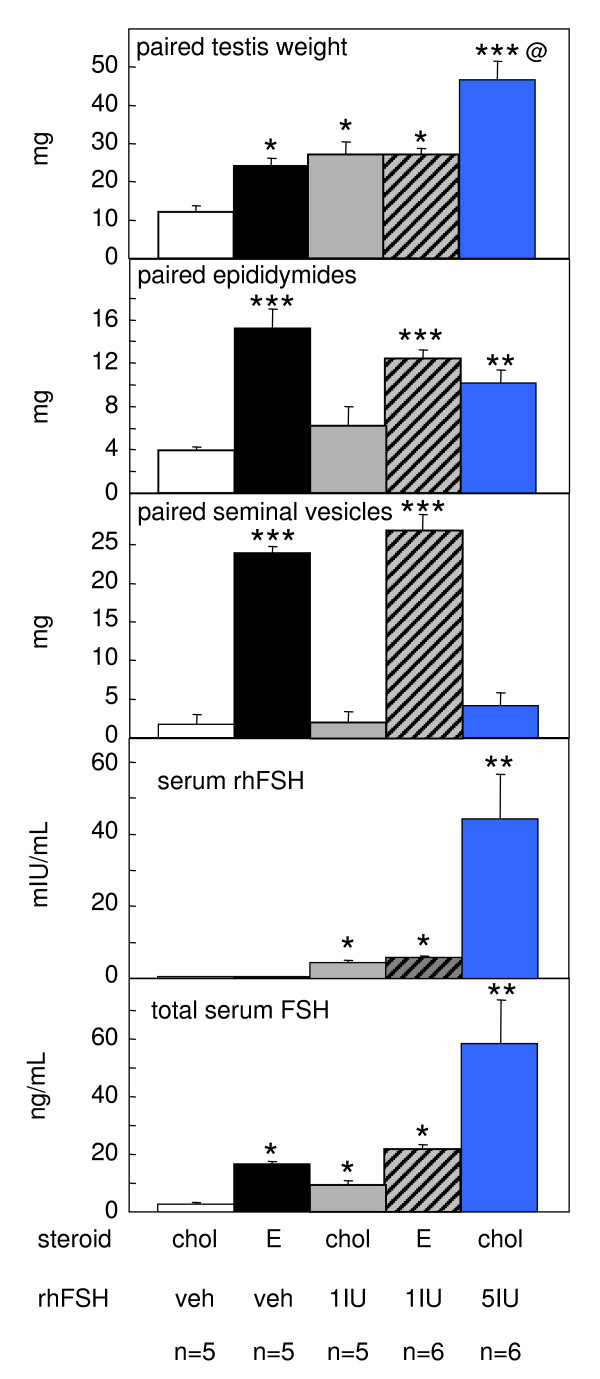
**Organ weights and serum FSH concentrations in *hpg *mice after treatment with estradiol and/or rhFSH**. Wet weight of paired testes (top), epididymides (second panel) and seminal vesicles (middle panel), and serum concentrations of FSH of male *hpg *mice as determined in the human FSH ELISA (fourth panel) and the rat RIA (bottom). Groups of mice were treated for 50 days with subcutaneous implants containing either cholesterol (chol) or 2% estradiol (E), and also daily treatment with vehicle, 1 or 5 U of recombinant human FSH (Gonal-F™). Values are group mean ± SEM, group sizes as indicated. *P < 0.05, **P < 0.01 and ***P < 0.001 vs group treated with cholesterol implants and vehicle; ^@^P < 0.01 vs all other groups.

Treatment with estradiol alone or with 1 IU rhFSH alone significantly (P < 0.05) increased testicular weight in *hpg *mice, as compared to the vehicle-treated controls (Fig. 3A). The combined estradiol plus 1 IU rhFSH treatment did not increase testis weight above that of the individual treatments (Fig. 3A). The high dose rhFSH treatment (5 IU) did produce a further increase in testis weight, such that testes weights were four-fold greater than the controls (P < 0.001; Fig. 3, top). The low dose (1 IU) rhFSH treatment produced no significant increase in epididymal weight (Fig. 3B), whereas in both the groups receiving estradiol (i.e. estradiol alone or estradiol with 1 IU rhFSH) there was a significant increase (P < 0.001, Fig. 3B). Treatment with 5 IU rhFSH produced a smaller increase in epididymal weight (Fig. 3B). Likewise, in both the groups receiving estradiol there was a large increase in the weight of the seminal vesicles (P < 0.001, Fig. 3C), whereas neither of the FSH treatments affected this organ (Fig. 3C).

### Serum FSH concentrations

Assay of serum samples using the human FSH kit confirmed that rhFSH was detectable in mice treated with rhFSH (Fig. 3D), and that the higher dose treatment (5 IU) produced a significantly higher circulating concentration of FSH (P < 0.01; Fig. 3D). There was significant crossreactivity of rhFSH in the rat RIA FSH assay so we were unable to measure endogenous mouse FSH directly in the animals treated with rhFSH. However, some inferences can be made. In the *hpg *mice receiving estradiol only, there was a significant increase (p < 0.05) in circulating FSH (Fig. 3E). Total serum FSH was also significantly higher (p < 0.05) in *hpg *mice receiving 1 IU rhFSH when compared to the control *hpg *group (Fig. 3E), and increased proportionately in the mice receiving 5 IU (Fig. 3E). In the *hpg *mice receiving both estradiol and 1 IU rhFSH there was also a proportionate increase in FSH compare to the *hpg *mice receiving estradiol only.

### Testicular histology

**Figure 4:**
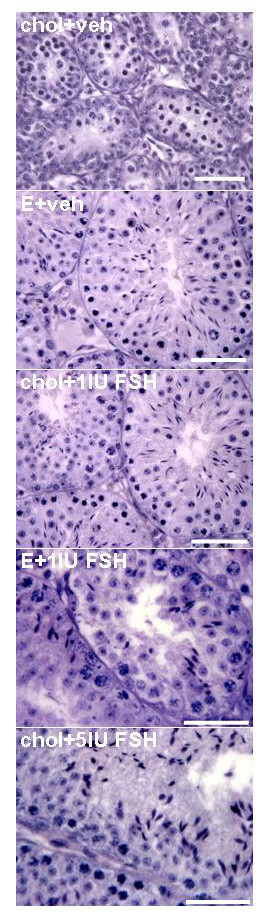
**Testis histology after treatment with estradiol and/or rhFSH**. Representative examples of testicular histology for *hpg *mice receiving subcutaneous implants containing either cholesterol (chol) or 2% estradiol (E), and also daily treatment with vehicle, 1 or 5 U of recombinant human FSH (Gonal-F™) for 50 days. Scale bars represent 50 μm.

**Figure 5:**
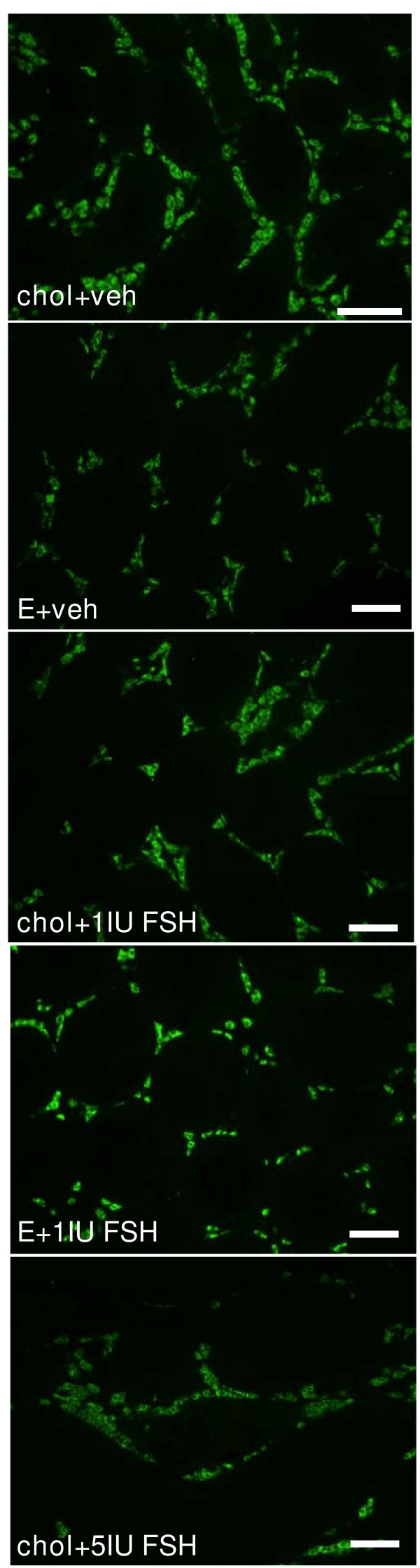
**Leydig cells in *hpg *mice after treatment with estradiol and/or rhFSH**. Representative examples of 3β-HSD immunostaining in *hpg *mice receiving subcutaneous implants containing either cholesterol (chol) or 2% estradiol (E), and also daily treatment with vehicle, 1 or 5 U of recombinant human FSH (Gonal-F™) for 50 days. Scale bars represent 50 μm.

**Figure 6:**
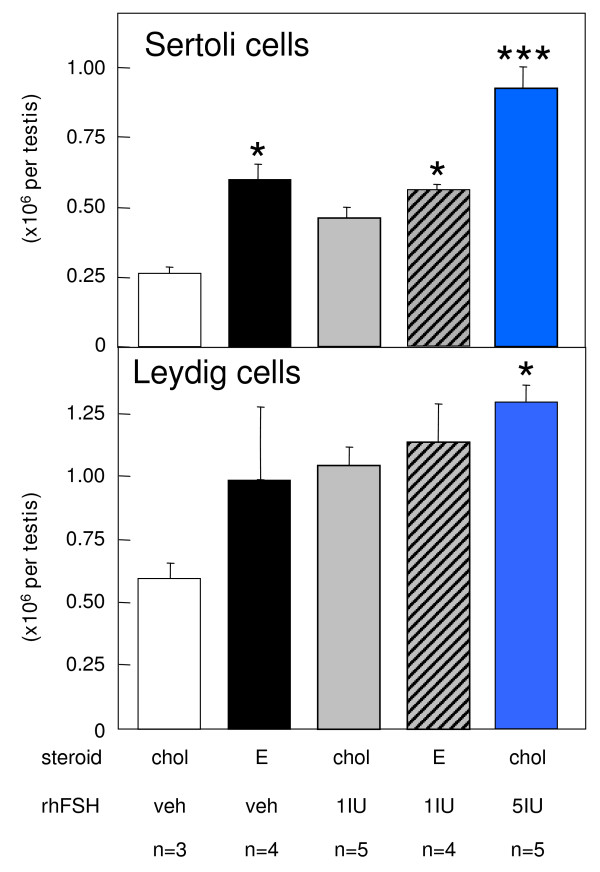
**Numbers of Leydig and Sertoli cells in *hpg *mice after treatment with estradiol and/or rhFSH**. Estimates of numbers of Leydig cells (top) and Sertoli cells (bottom) in testes of *hpg *mice that had been treated for 50 days with subcutaneous implants containing either cholesterol (chol) or 2% estradiol (E), and also daily treatment with vehicle, 1 or 5 U of recombinant human FSH (Gonal-F™). Values are group mean ± SEM, group sizes as indicated. *P < 0.05 and ***P < 0.001 vs group treated with cholesterol implants and vehicle.

Testes from *hpg *mice that were treated with cholesterol implants and vehicle had a characteristic undeveloped appearance, with small-diameter seminiferous tubules lacking a lumen (Fig. 4, Table 1) and Sertoli cells frequently located close to the basal lamina (Fig. 4). Round and elongating spermatids were not observed and germ cell development in this control group did not develop past the primary spermatocyte stage. Testicular sections from all the other groups of *hpg *mice treated with estradiol or rhFSH revealed that the increase in testicular weight was a reflection of development of the seminiferous epithelium, and presence of lumina (Fig. 4, Table 1). Elongating spermatids were present in the majority of tubules, though these were more abundant in tubules in the group treated with the higher dose of FSH (Fig. 4). Morphometric analysis revealed that both estradiol alone and 1 IU/day rhFSH alone increased the volume of the seminiferous epithelium and thus the total tubule volume compared to the cholesterol+vehicle treated control *hpg *mice (Table 1). The combined estradiol + 1 IU/day rhFSH also significantly increased these parameters compared to the controls, but this combined treatment did not increase significantly any parameter of tubule development compared to *hpg *mice receiving either of the treatments alone (Table 1). Daily treatment with 5 IU rhFSH did produce significantly greater increases in seminiferous epithelium volume, tubule volume and total tubule length as compared to the separate or combined estradiol and 1 IU/day rhFSH treatments (Table 1). The 3β-HSD immunostaining revealed that Leydig cells were abundant in the interstitial compartments of the testes from *hpg *mice (Fig. 5), but only the daily treatment with 5 IU rhFSH produced a significant increase in Leydig cell numbers (Fig. 6, lower panel). In contrast, treatment with estradiol alone significantly increased the number of Sertoli cells (Fig. 6, upper panel), as did treatment with the higher dose of rhFSH, as did the combined treatment of estradiol plus 1 IU rhFSH/day (Fig. 6).

**Table 1:** Morphometric analyses of testes in *hpg *mice after treatment with estradiol and/or rhFSH

	cholesterol vehicle	estradiol vehicle	cholesterol 1 IU FSH	estradiol 1 IU FSH	cholesterol 5 IU FSH
N	3	4	4	5	5
body wt (g)	29.7 ± 3.2	30.0 ± 2.5	29.5 ± 2.5	29.8 ± 2.8	29.0 ± 3.2
testis wt (mg)	12.0 ± 0.1	24.2 ± 1.8^a^	27.2 ± 3.3^a^	27.2 ± 1.7^a^	46.6 ± 4.8^abc^
tubule diameter (μm)	50.3 ± 0.7	71.6 ± 3.3^a^	66.3 ± 1.6^a^	72.3 ± 2.2^a^	77.3 ± 3.5^a^
tubule volume (μl)	3.8 ± 0.7	11.2 ± 1.1^a^	12.1 ± 0.39	12.2 ± 1.1^a^	21.8 ± 2.3^abc^
tubule length (m)	2.2 ± 0.4	2.8 ± 0.2	3.3 ± 0.2	3.0 ± 0.2	4.6 ± 0.3^abc^
tubule (% testis)	76.9 ± 9.9	91.3 ± 1.4	92.5 ± 0.8^a^	87.9 ± 1.5	94.0 ± 0.7^a^
sem epith vol (μl)	3.6 ± 0.6	9.9 ± 0.9^a^	11.0 ± 0.2^a^	10.5 ± 0.9^a^	19.4 ± 2.0^abc^
lumen volume (μl)	0.1 ± 0.1	1.3 ± 0.3	1.6 ± 0.3	1.1 ± 0.1	2.4 ± 0.4

Values are group mean ± SEM, n = number of mice where complete stereological analysis was carried out. ^a^P < 0.05 vs cholesterol + vehicle, ^b^P < 0.05 vs estradiol + vehicle, ^c^P < 0.05 vs cholesterol + 1 IU FSH. sem epith vol = seminiferous epithelium volume

## Discussion

The first objective of these studies was to confirm the expression of ERα receptors in the pituitary gland and testis of *hpg *mice. Complementary immunohistochemical and Western blot studies revealed that ERα immunoreactivity is present in both the pituitary and testis of *hpg *mice at levels comparable to that in wild-type litter mates. The immunohistochemical studies demonstrated that the distribution of ERα in the testis of the *hpg *mouse was restricted to putative Leydig cells in the interstitium and peritubular myoid cells, consistent with previous studies in the mouse [23]. Interestingly, we also observed ERα immunoreactivity in angular nuclei adjacent to the basal lamina in testes from wild-type littermates, these are very likely to be Sertoli cells. Previous studies in mice have not reported ERα expression in this cell type [23]. Our observations suggest that estradiol could potentially act via ERα in both the pituitary and testis in the *hpg *mouse. We were unable to confirm using immunohistochemistry whether ERβ is also expressed in the testis of the *hpg *mice due to technical limitations of the antiserum, but Western blots did reveal protein bands of the appropriate molecular weight of ERβ in tissues from both *hpg *and wild-type mice (data not shown). Previous studies have provided rather conflicting evidence for ERβ expression in the mouse testis, some studies demonstrating ERβ expression in the Leydig cells and elongating spermatids of adult mice [24], others reporting a developmental decline in ERβ expression in the mouse testis, with immunoreactivity being undetectable in the adult [25]. As ERβ expression does not appear to be required for normal testis function in the mouse [26], our studies have focussed on the role of the ERα receptor.

We again observed robust stimulatory effects of estradiol treatment on the testis in *hpg *mice, consistent with our previous studies [9,15,16]. The current study revealed that these effects of estradiol were both qualitatively and quantitatively very similar to the effects of treatment with FSH. It is well established that treatment with FSH alone will induce testicular development in *hpg *mice [14], though we observed a greater degree of development. In our study, both doses of rhFSH induced germ cell development up to the elongating spermatid stage and the also the formation of small lumina in the tubules, whereas Singh and Handelsman [14] reported that the same doses of rhFSH applied for the same duration (50 days) mainly stimulated premeiotic germ cell proliferation, and that relatively few cells progressed to the round spermatid stage. We observed a significant increase in numbers of Sertoli cells after estradiol and high dose rhFSH treatment, which Singh and Handelsman [14] did not detect. Given that the estradiol treatment clearly elevated circulating FSH concentrations, this observation is consistent with the key role of FSH in Sertoli cell proliferation in mice [27], and with more recent studies where genetic induction of FSH production in *hpg *mice has increased Sertoli cell production [28]. We also observed an increased number of Leydig cells in *hpg *mice treated with the high dose of FSH. Stimulatory actions of FSH on Leydig cell activity and morphology have been reported previously in *hpg *mice and are likely to reflect indirect actions mediated by paracrine output from Sertoli cells [29]. Unfortunately insufficient serum was available to measure circulating testosterone concentrations after the various experimental treatments as we considered it to be much more important to measure endogenous and recombinant FSH in the serum that we collected in order to verify the efficacy of our treatment protocols. In our previous studies we have been unable to measure detectable increases in either serum testosterone [9,15] or in intratesticular testosterone [15] after estradiol treatment, but on the basis of previous studies in *hpg *mice it seems likely that the FSH treatments would have increased testicular androgen production [29].

Though model-based, our estimates of cell numbers are consistent with those obtained by ourselves and others using more robust, design-based stereological procedures [9,27,30,31]. An important difference between the current study and that of Singh and Handelsman [14] is that in the latter study the 50-day rhFSH treatment was initiated at a juvenile age (day 21), whereas we treated *hpg *mice at an "adult" age. We have previously observed that there is a degree of spontaneous testicular development in *hpg *mice probably reflecting a low level of GnRH-independent production of gonadotropins [1]. Thus, our experimental rhFSH treatments may well have been superimposed upon a marginally higher endogenous level of testicular development or androgen production, and given the key role for androgens in murine spermatogenesis [13,15] this seems a highly likely explanation for the differences between the current and the previous study.

The key outcome of the current studies is that the combined estradiol and rhFSH did *not *produce a greater degree of stimulation of the seminiferous epithelium that treatment with either hormone alone. This is contrary to our initial prediction, and to studies in the neonatal rat where combined treatment with estradiol benzoate and purified human FSH produce effects on development of the seminiferous epithelium not seen after treatment with either hormone alone, including increased tubule diameters, induction of a lumen and increased numbers of pachytene spermatocytes [32]. The observation that treatment with a higher dose of rhFSH (5 IU daily) did increase the volume of the seminiferous epithelium and tubules further demonstrates that the lack of additive or synergistic effect is not a consequence of maximal proliferation rates already being achieved. Given the qualitative and quantitative similarity of the effects of estradiol and rhFSH that we observed, we conclude that the primary action of estradiol on testis function is indirect, via the stimulation of FSH release, in the *hpg *mouse experimental model. Although this stimulatory effect of estradiol may seem like a paradoxical response in a male mammal, it was demonstrably the case that circulating FSH levels were increased in this and previous studies in the *hpg *mouse. This paradoxical effect in the male is not due to incomplete masculinization of the *hpg *mouse [16], rather it appears to be a reflection of the fact that development of the reproductive axis in the *hpg *mouse is arrested at a neonatal stage [1,11] when steroid negative feedback pathways have not yet developed. It is worth noting that certain actions of estradiol on the male reproductive tract in mice *are *highly likely to be direct actions. In the current study we observed that estradiol treatment produced a substantial increase in the wet weight of the seminal vesicles, whereas FSH had no effect on this tissue nor did it augment the actions of estradiol. This is consistent with a previous study in the *hpg *mouse [9], which also provided evidence for direct effects of estradiol on the prostate gland [9].

Recent evidence points to an autocrine or paracrine role for estradiol in the testis during fetal and early neonatal development whereby it inhibits production of androgens by Leydig cells [33]. Perhaps the endocrine actions of estradiol revealed in the *hpg *mouse represent a physiological role in co-ordinating early development. Whilst estradiol production acts within the testis to retard early androgen production, it also acts in the pituitary gland to stimulate FSH production and thereby promote early Sertoli cell proliferation. Thus, when pubertal increases in LH production eventually occur and thereby stimulate androgen production, the somatic cell complement within the tubules has already been established and will support the androgen-driven spermatogenesis.

## Competing interests

The author(s) declare that they have no competing interests.

## Authors' contributions

HB carried out the tissue processing and stereological analyses on the seminiferous tubules, MOW carried out the in vivo studies and the Western Blot and ER immunohistochemistry, GRH and RAW carried out the immunostaining for 3β-HSD and the quantitative assessment of Leydig and Sertoli cell numbers, TMM developed the stereological procedures and oversaw their application, FJPE managed the project and wrote and revised the manuscript.
